# Current Practice in the Referral of Individuals with Suspected Dementia for Neuroimaging by General Practitioners in Ireland and Wales

**DOI:** 10.1371/journal.pone.0151793

**Published:** 2016-03-23

**Authors:** Aurelia S. Ciblis, Marie-Louise Butler, Catherine Quinn, Linda Clare, Arun L. W. Bokde, Paul G. Mullins, Jonathan P. McNulty

**Affiliations:** 1 School of Medicine, University College Dublin, Dublin, Ireland; 2 School of Psychology, Bangor University, Bangor, United Kingdom; 3 School of Psychology, University of Exeter, Exeter, United Kingdom; 4 Institute of Neuroscience, Trinity College Dublin, Dublin, Ireland; University Of São Paulo, BRAZIL

## Abstract

**Objectives:**

While early diagnosis of dementia is important, the question arises whether general practitioners (GPs) should engage in direct referrals. The current study investigated current referral practices for neuroimaging in dementia, access to imaging modalities and investigated related GP training in Ireland and North Wales.

**Methods:**

A questionnaire was distributed to GPs in the programme regions which included approximately two thirds of all GPs in the Republic of Ireland and all general practitioners in North Wales. A total of 2,093 questionnaires were issued.

**Results:**

48.6% of Irish respondents and 24.3% of Welsh respondents directly referred patients with suspected dementia for neuroimaging. Irish GPs reported greater direct access to neuroimaging than their Welsh counterparts. A very small percentage of Irish and Welsh GPs (4.7% and 10% respectively) had received training in neuroimaging and the majority who referred patients for neuroimaging were not aware of any dementia-specific protocols for referrals (93.1% and 95% respectively).

**Conclusions:**

The benefits of direct GP access to neuroimaging investigations for dementia have yet to be established. Our findings suggest that current GP speciality training in Ireland and Wales is deficient in dementia-specific and neuroimaging training with the concern being that inadequate training will lead to inadequate referrals. Further training would complement guidelines and provide a greater understanding of the role and appropriateness of neuroimaging techniques in the diagnosis of dementia.

## Introduction

The majority of people with dementia currently do not receive a formal diagnosis [1–3]. Only 20–50% of dementia cases in high income countries are recognised and documented in primary care, and this ‘treatment gap’ is even greater in low and middle income countries, with a study in India suggesting that 90% of cases remain unidentified [1]. In the UK, approximately 44.2% of people with dementia are formally diagnosed and the situation is similar in Ireland [4,5]. Though there is at present no cure for dementia, this disparity is a significant barrier to improving lives of people with dementia and their carers [1]. A specific diagnosis affects treatment decisions [6] and early diagnosis of dementia is crucial since some treatments are more effective in the early stages, and earlier diagnosis and timely intervention provide health, financial and social benefits [1]. In addition, people with undiagnosed dementia generally have no access to the treatment, care and organised support that formal diagnosis provides, and they cannot avail of currently available interventions which can be efficient in improving symptoms [1].

Neuroimaging is increasingly regarded as an essential part of the investigation of a patient with suspected dementia, and some guidelines recommend the use of structural imaging in the evaluation of every suspected dementia case [7–9]. The use of structural imaging is recommended to exclude other cerebral pathologies including neoplasm, normal pressure hydrocephalus, infarction, haemorrhage and haematomas, and to help establish a subtype diagnosis [8,9]. Magnetic resonance imaging (MRI) is the preferred neuroimaging modality to assist with early diagnosis and detect subcortical vascular changes, however, computed tomography (CT) scanning can also be used while nuclear medicine scans are generally employed to differentiate between various dementia subtypes [8,9].

General practitioners (GPs) play a key role in the diagnosis of dementia [2,10,11]. GPs work at the frontline of the healthcare system and are the first point of access to identify patients with suspected dementia, initiate a diagnostic evaluation, and make referrals [12,13] and are thus important gatekeepers [1,7]. In the UK, GPs are financially rewarded for identifying and registering people with a dementia diagnosis and subsequently monitoring their care [14] and current UK guidelines recommend that GPs perform a comprehensive assessment of patients with suspected dementia [15]. The next step in the diagnostic process consists of the exclusion of possible reversible conditions and a subtype diagnosis which generally includes the use of neuroimaging and referral to a specialist in secondary care or a memory clinic [7].

In the recently launched National Dementia Strategy in Ireland, timely diagnosis and intervention has been identified as a priority area [16]. This strategy listed the development of dementia-specific reference materials for GPs and the development of guidance on national and local diagnostic pathways and access to diagnostic services for GPs [16]. Unlike the UK, Ireland has no national clinical guidelines for dementia. A diagnosis of dementia is generally made through primary care services [17]. Similar to the UK, guidelines by the Irish College of General Practitioners (ICGP) recommend that GPs conduct appropriate investigations [13]. A referral to specialist services is preferred to confirm the diagnosis, exclude other pathologies, and establish a subtype diagnosis. Memory clinics are increasingly being established as specialist centres for differential diagnosis. They are not available in every health service area, however, and there is substantial variability in the kind of service they offer and how they are resourced [13,18].

It is therefore important to note that the healthcare systems in Ireland and the UK differ. In the UK, the overwhelming majority of GPs work under the public healthcare system, the National Health Service (NHS), and about 11% of the population has some form of private medical insurance [19]. In Ireland, GPs work either in private practice, under the general medical scheme (GMS), or in mixed practices consisting of public and private patients, and approximately 45% of the population have private health insurance [20]. There is a lack of accurate, complete and comparable information about patient referral pathways in Ireland [14]. Some GPs in Ireland avail of direct access to radiological services, though waiting times for investigations vary greatly between public and private patients [21]. To our knowledge, there are currently no data available on the number of direct GP referrals for neuroimaging for suspected cases of dementia. Moreover, the incremental value of neuroimaging from primary care is unknown [11].

According to Alzheimer’s Disease International recommendations, all primary care services should have basic competency in early detection of dementia, making and imparting a provisional dementia diagnosis, and initial management of dementia [1]. However, a study by the Alzheimer’s cooperative valuation in Europe (ALCOVE) involving 27 EU countries revealed that 70% of countries reported that family doctors had inadequate training in the diagnosis of dementia and recognising symptoms of early dementia [22]. In a UK study of 8,051 GPs, less than half of the respondents reported that they had received sufficient training to help them diagnose and manage dementia [23]. Research in Ireland similarly suggests that GPs feel under-skilled in the diagnosis and treatment of dementia and are keen on training and guidance in the area [24,25]. In a 2012 opinion piece, O’Connell suggests to enable the diagnosis of most cases of dementia, it is imperative that GPs are adequately trained in clinical assessment skills and access to key diagnostics [10]. Since diagnosis at an earlier stage increases the complexity of the diagnostic process, it has been suggested that this complexity is addressed by non-specialists if people are to be diagnosed early [22].

While early diagnosis of dementia is important, the question arises whether GPs should engage in direct referrals for neuroimaging considering the lack of knowledge and training in the area [21]. Our earlier work focused on Irish GPs, explored urban versus rural differences, and the relevance and role of the formal radiology report for GPs [26]. In order to address outstanding issues, the current study investigated GPs’ current referral practices for neuroimaging in dementia in Ireland and North Wales. It explored their access to different imaging modalities in view of existing guidelines and investigated their training in the area. The findings provide an insight into current GP practice regarding neuroimaging in dementia and can thus help to inform future decisions on training and service provision.

## Methods and Materials

### Study design and participants

The research employed a questionnaire-based study design. The survey was completely anonymous and targeted GPs with practices located within the European Territorial Cooperation (better known as INTERREG) Ireland-Wales programme area (a specific programme area under the European Regional Development Funding) (Table 1), which included approximately two thirds of all GPs in the Republic of Ireland as identified through the Irish Medical Directory (IMD) 2011–2012 (as described in Ciblis et al. [26]), and all general practitioners in North Wales as identified through the Welsh health board website (www.wales.nhs.uk). A total of 2,093 questionnaires were issued by post to all 1,684 GPs within the Irish counties making up the Ireland-Wales programme area and a further 409 questionnaires were sent by post and email to the GPs in North Wales within the Welsh counties in the Ireland-Wales programme area. No reminders were issued.

**Table 1:** Counties included in the European Regional Development Fund INTERREG Ireland Wales programme area.

Ireland	Wales
Carlow, Dublin, Kildare, Kilkenny, Meath, South Tipperary, Waterford, Wexford, Wicklow and the adjacent counties Cork and Kerry.	Anglesey, Conwy, Denbighshire, Flintshire, Gwynedd, and Wrexham.

This study was approved by the Bangor University School of Psychology Ethics and Research Committee (reference: 2013–8364) and received an exemption from full institutional ethical approval from the University College Dublin Human Research Ethics Committee—Life Sciences (reference: LS-E-12-191-McNulty).

### Materials

The questionnaire was developed in collaboration with dementia specialists. Following an initial discussion on the purpose of the questionnaire all authors, together with collaborators, contributed to the content and design of the questionnaire which then underwent a three phase review. This consisted of an initial review of the draft questionnaire by the authors followed by a review by external collaborators including dementia specialists, radiologists and GPs. Finally a pilot of the questionnaire using GPs affiliated with the collaborating institutions and on several researchers at one of the research centres was completed.

The questionnaire consisted of five main sections and included open and closed questions. Section A ascertained the demographic characteristics of the sample. Section B enquired about satisfaction with diagnostic capabilities within the health service as well as access to neuroimaging. Section C established specialists’ current referral patterns in neuroimaging in dementia, their reasons for referral as well as their use of protocols. Section D asked about the usefulness of reports on neuroimaging investigations. Section E enquired about knowledge and training in neuroimaging and dementia (S1 File).

### Statistical analysis

Data analysis was carried out using IBM SPSS Statistics Version 20. Descriptive statistics are reported for most variables. Content analysis was used to examine open questions.

## Results

### Demographics

A total of 302 questionnaires were returned from Irish GPs and 72 questionnaires from Welsh GPs, corresponding to a response rate of 17.9% and 17.6%, respectively. Irish respondents’ year of qualification ranged from 1965–2012 with 32.7% qualifying in 1975–1984, 26.9% in 1985–1994, and 18.5% in 1995–2004. Welsh respondents’ year of qualification ranged from 1974–2009, with 34.9% qualifying in 1975–1984 and 37.2% in 1985–1994. The average number of years participants had spent in their current job was 18.2 years (SD = 11.47; range = 0.5–44) for Irish GPs and 16.2 years (SD = 9.15; range = 1–32) for Welsh GPs.

Just over half of Irish participants’ practices (55.8%) were located in urban areas and almost a third (29.3%) in semi-rural locations (i.e. towns with a population of 1,500–5,000). The majority of Welsh respondents’ surgeries (69%) were located in semi-rural areas; only 16.9% were located in rural and just 14.1% in urban areas. Most Irish GPs (88.3%) worked in mixed practices, i.e. in private practice and under the Irish GMS which entitles patients with an income below a particular figure to receive certain health services free of charge, whereas all Welsh GPs worked in the public health service.

A total of 44.8% of Irish respondents reported that between 25–49% of their patients were aged over 65 years. Similarly, 50% of Welsh respondents reported that 25–49% of their patients was aged over 65 years. Nearly all Irish participants (94.7%) and 88.7% of Welsh respondents were involved in the diagnosis of mild cognitive impairment (MCI) or dementia.

### Diagnostic capabilities within the health service area

The majority of Irish respondents rated the proficiency in the diagnosis of MCI or dementia within their Health Service Executive (HSE) region as excellent (11.5%) or good (49.7%), however, over a third (34.1%) rated them as fair and 4.7% as poor. Welsh respondents rated the same proficiency within their health board area less favourably, with only 5.7% stating it was excellent and 45.7% rating it as good, while nearly half rated it as fair (40%) or poor (8.6%).

Better access to specialists and services, such as memory clinics and imaging, as well as more staff were mentioned as ways to increase the number of people who receive a diagnosis of MCI or dementia.

### Importance of neuroimaging for the diagnosis of dementia

**Fig 1:**
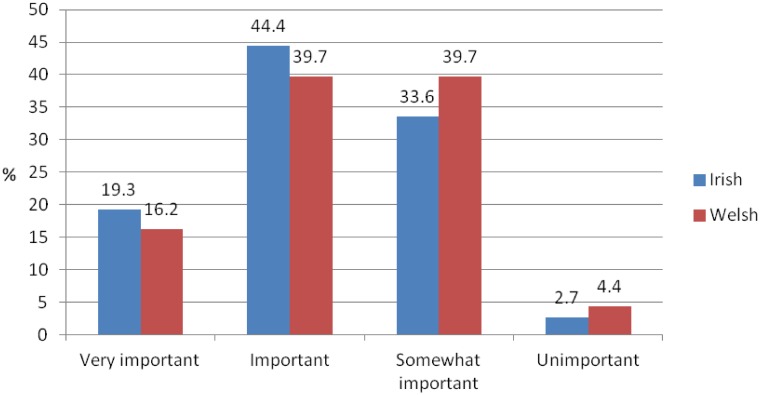
Importance of neuroimaging for the diagnosis of dementia.

Irish GPs considered neuroimaging for the diagnosis of dementia as opposed to other diagnostic tests more important than Welsh GPs (Fig 1).

### Current referrals for neuroimaging

Just under half of Irish respondents (48.6%) referred patients with suspected dementia for neuroimaging, and 41.2% referred patients with suspected MCI. This compared to only 24.3% of Welsh respondents who referred patients with suspected dementia for neuroimaging and 14.3% who referred patients with suspected MCI. Of those respondents who referred these cases for neuroimaging, 75.6% of Irish as opposed to 31.6% of Welsh respondents referred for MRI; 71.8% of Irish respondents referred for CT compared to 63.2% of Welsh GPs, and only one Irish and one Welsh participant reported that they referred such cases for nuclear medicine imaging (several modalities could be selected).

### Access to neuroimaging

**Fig 2:**
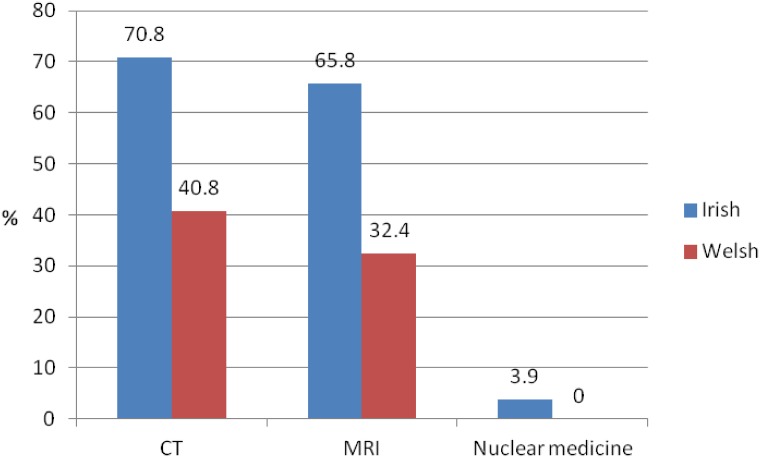
Reported availability of neuroimaging modalities for direct referral.

GPs’ reported access to neuroimaging for direct referral is displayed in Fig 2. Irish GPs reported better availability of various neuroimaging modalities for direct referral overall. The percentage of Irish GPs who reported access to MRI was nearly twice as high as the percentage of Welsh GPs. Nuclear medicine examinations such as 2-[18F]fluoro-2-deoxy-D-glucose positron emission tomography (FDG-PET) or FDG-PET/CT, perfusion hexamethylpropyleneamine oxime (HMPAO) single-photon emission computed tomography (SPECT) or dopaminergic iodine-123-radiolabelled 2β-carbomethoxy-3β-(4-iodophenyl)-N-(3-fluoropropyl) nortropane (FP-CIT) SPECT were available to only 3.9% of Irish GPs and were not available to the Welsh respondents.

A total of 50.8% of Irish and 33.3% of Welsh respondents who referred patients for neuroimaging reported that there were modalities that they would like to have access to but that were unavailable to them. These were mainly CT and MRI through the public healthcare system. Traditionally in Ireland and the UK, public patients generally require a referral to secondary or tertiary care to access neuroimaging whereas private patients can often avail of direct access, however, this is changing with direct access now becoming available to GPs in many centres across the UK.

### Reasons for referral

**Fig 3:**
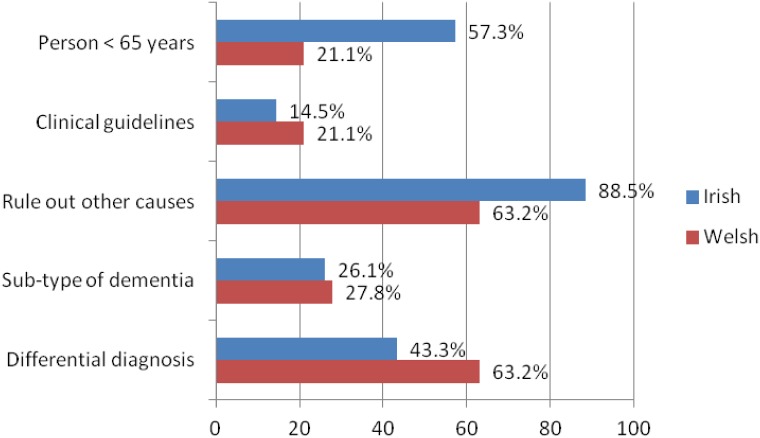
Reported reasons for referral for neuroimaging.

The majority of Irish GPs referred patients with suspected MCI or dementia to rule out other causes (Fig 3). Likewise, most Welsh GPs referred for this reason, however, an equal percentage referred to establish a differential diagnosis. Considerably more Irish GPs referred on the basis that the patient was less than 65 years of age. More Welsh than Irish respondents based the referral on clinical guidelines such as the guidelines by the National Institute for Health and Clinical Excellence / Social Care Institute for Excellence [7].

**Fig 4:**
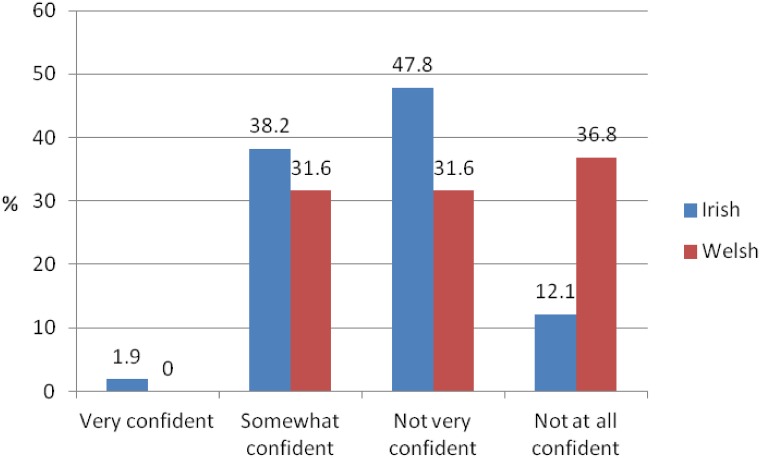
Confidence in selecting the most appropriate neuroimaging modality.

Confidence in the selection of the appropriate neuroimaging modality Although more or less than a third of Irish and Welsh GPs were somewhat confident in selecting the most appropriate neuroimaging modality, nearly half of Irish GPs (47.8%) were not very confident and more than a third of Welsh GPs were not at all confident in selecting the most appropriate neuroimaging modality (Fig 4).

The majority of Irish and Welsh GPs who referred patients with suspected MCI or dementia for neuroimaging were not aware of any dementia-specific protocols for referrals (93.1% and 95%, respectively).

### Training

**Fig 5:**
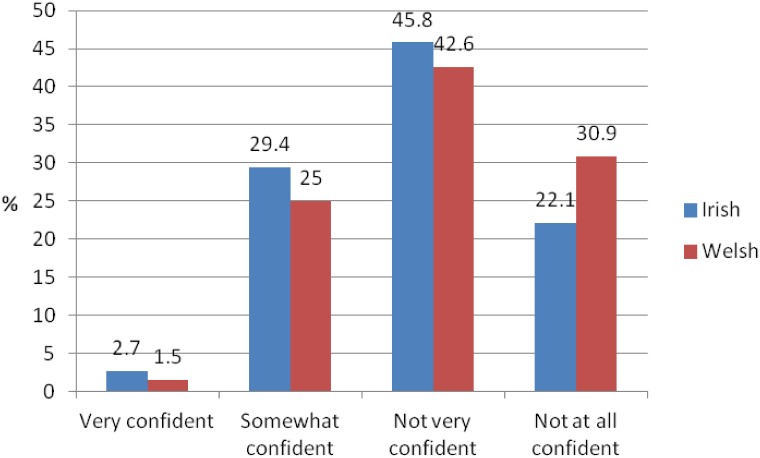
Confidence in understanding of neuroimaging in dementia.

More than a third of Welsh (34.8%) compared to over a fifth Irish respondents (22.5%) reported that they had received any form of dementia-specific training, and only 10% of Welsh GPs and just 4.7% of Irish had received any form training on neuroimaging. Less than a third of Irish respondents were somewhat confident in their understanding of neuroimaging in dementia, and the majority were not very confident or not at all confident (Fig 5). Similarly, only a quarter of Welsh respondents were somewhat confident in in their understanding of neuroimaging in dementia, while most were not very confident or not at all confident.

**Fig 6:**
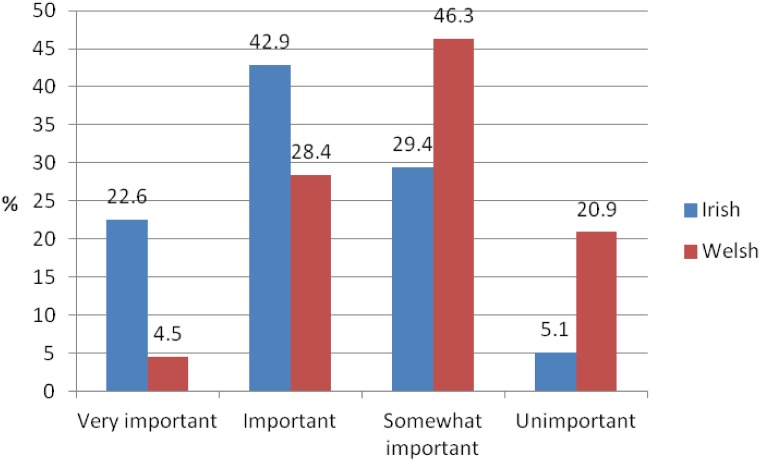
Importance of furthering knowledge about neuroimaging in dementia.

A higher percentage of Irish (89.1%) compared to Welsh (66.2%) respondents reported that they would like to receive more information about the different types of neuroimaging available. Most Irish GPs found it important (42.9%) or very important (21.6%) to further their knowledge about neuroimaging whereas only 26.9% of Welsh respondents found it important, and nearly half (47.8%) found it only somewhat important. Similarly, just over a quarter of Welsh respondents found it important to further their knowledge about neuroimaging in dementia, and nearly half found it somewhat important compared to over a fifth of Irish respondents who found it very important and over 42.9% who found it important (Fig 6).

While over half of Irish respondents were interested in training in neuroimaging (52.9%) or neuroimaging in dementia (56.1%), only 40% of Welsh respondents were interested in training in neuroimaging and 43.3% in neuroimaging in dementia.

## Discussion

The current study revealed that almost half of Irish respondents and a quarter of Welsh respondents directly referred patients with suspected dementia for neuroimaging. Yet reported levels of confidence and training in the area were found to be very low. Only about a third of GPs were somewhat confident in selecting the most appropriate neuroimaging modality, and the majority of respondents were only slightly or not at all confident in understanding neuroimaging in dementia. A very small percentage of respondents had received training in neuroimaging (within the radiology department, as part of general training or as part of continuing medical education), and only about a fifth of Irish and a third of Welsh GPs had received dementia-specific training. Similar to the current findings, a survey among Irish GPs in 2006 found that the majority of respondents (90%) had no dementia-specific training [24]. In two UK studies just half of GPs agreed that they had received sufficient basic and post-qualifying training to help them diagnose or manage dementia [23,27] while a survey by the Alzheimer’s Society (UK) revealed that only 37% of GPs felt that they had received adequate basic training on dementia [28]. The results of the current study suggest that GPs have an interest in furthering their knowledge about neuroimaging in dementia, however, the interest was less pronounced among Welsh GPs, possibly due to the fact most referred patients with suspected dementia to memory clinics and not directly to neuroimaging. Such a referral approach, particularly for specialist imaging examinations, is recommended in order to minimise inappropriate imaging referrals, to improve consistency in referrals and modality selection, independent of the geographical location of the GP [7,10,13]. This requires the involvement of the GP, specialist and radiologist, however, the barrier of access to services based on geographic location or health insurance status remains problematic.

In view of the self-reported lack of knowledge and training in neuroimaging in dementia the question arises whether GPs should directly refer patients with suspected dementia for neuroimaging. The benefits of direct GP referral for such investigations have not been established and further research into this topic is warranted [29]. There are currently no national guidelines to assist Irish GPs in the diagnosis of dementia [5]. Likewise there are no national referral guidelines for access to radiological diagnostics although the introduction of such guidelines has been recommended by the ICGP [21]. Although GPs in the UK avail of the NICE/SCIE guidelines, only a fifth of Welsh respondents in this study based their referral of suspected dementia cases for neuroimaging on this guidance. Research on the use of cognitive screening instruments in primary care following the introduction of the NICE/SCIE guidance and the national dementia strategy showed that use of such instruments did not increase significantly in spite of recommendations for their use suggesting that there might be a problem with the adherence to guidelines and that further support on their implementation might be required [30]. Thus highlighting that guidelines and recommendations alone may not be sufficient to change current practices and make the best use of resources, and emphasising the importance of associated further education and training.

Inconsistencies regarding the adherence to the NICE/SCIE guidance are also reflected in the fact that most Welsh GPs who referred suspected cases of dementia for neuroimaging referred these patients for CT which was also the most available modality. Thus suggesting that referral is based on availability rather than training as structural MRI is the preferred modality to assist with early diagnosis and the detection of subcortical vascular changes according to the NICE/SCIE guidelines [7]. Though the referral to CT might be based on difficulties in accessing resources, it could also point to the above mentioned lack of knowledge of neuroimaging in dementia and might have an impact on patient diagnosis and constitute an incorrect use of resources.

Nearly twice as many Irish GPs compared to Welsh GPs reported that they referred suspected cases of dementia for neuroimaging. Differences in the two healthcare systems are the likely reason. In both countries, patients with suspected dementia are generally first recognised in primary care and subsequently referred to secondary care services as outlined above. Since all Welsh participants in the current study worked in the public health service, they were more likely to refer patients to memory clinics, whereas the majority of the Irish GPs worked in mixed practices which allowed them to refer private patients directly for neuroimaging. This raises the question of the appropriateness of different referral pathways for public and private patients. In light of GPs’ self-stated deficient knowledge and training in neuroimaging in dementia and a lack of clear guidance in the area, direct GP referral for private patients might therefore not be appropriate.

In addition to the difference in the healthcare systems, geographical differences between the two countries are likely to have impacted upon the reported availability of the various imaging modalities as well as the rating of the diagnostic capabilities in the diagnosis of dementia within the health service area. While over half of Irish respondents’ practices were located in urban areas this applied to just over ten per cent of respondents in North Wales. Urban areas tend to offer increased service provision [31]. The urban rural divide might have contributed to the finding that Welsh GPs had reduced access to MRI when compared to Irish GPs. This might have an impact on diagnostic accuracy and might reflect that people with dementia living in rural areas might be disadvantaged [5].

Respondents from both countries reported extremely restricted access to nuclear medicine imaging. Such access might not be needed for the routine investigation of dementia, however, since it is generally only required for the establishment of a subtype diagnosis which should be made by healthcare professionals with expertise in differential diagnosis according to the National Institute for Health and Clinical Excellence / Social Care Institute for Excellence (NICE/SCIE) guidelines [7].

The reasons for patient referrals for imaging differed between Irish and Welsh GPs. A higher proportion of Irish GPs compared to Welsh GPs referred patients with suspected MCI or dementia for neuroimaging to rule out other causes, while a higher fraction of Welsh GPs referred to establish a differential diagnosis. Over half of Irish GPs compared to only a fifth of Welsh GPs referred suspected dementia cases for neuroimaging because the patient was less than 65 years of age. Since there are demographic differences between the two areas with North Wales having a higher proportion of people aged over 65 years than Wales as a whole while Ireland has a younger population, Irish GPs might have referred patients with suspected early onset dementia more frequently which would also in part explain their higher reported level of referrals to rule out other causes [32–34].

The majority of Irish and Welsh respondents regarded neuroimaging for the diagnosis of dementia as opposed to other diagnostic tests as important or very important, and many GPs stated an interest in training in the area. In view of the current lack of training and knowledge in the area, the provision of neuroimaging for the diagnosis of dementia might be better served through referrals from specialists in secondary care. Our findings suggest that current GP speciality training in Ireland and Wales is deficient in dementia-specific and neuroimaging training with the concern being that inadequate training will lead to inadequate referrals.

## Conclusion

In view of the vast under-diagnosis of dementia, a comprehensive approach to dementia diagnosis is required. The benefits of direct GP access to neuroimaging investigations for dementia have yet to be established and require further investigation. Further training in neuroimaging would complement guidelines and provide GPs with a greater understanding of the role and appropriateness of neuroimaging techniques in the diagnosis of dementia.

## Supporting Information

S1 File Full Questionnaire. (DOC)
