# Supplementary material for: Current Practice in the Referral of Individuals with Suspected Dementia for Neuroimaging by General Practitioners in Ireland and Wales
Source: PLoS One. 2016 Mar 23;11(3):e0151793. doi: 10.1371/journal.pone.0151793 (PMC4805299; doi:10.1371/journal.pone.0151793)
Supplement: S1 File — (DOC) [file pone.0151793.s001.doc]

**Confidential Questionnaire**

**Neuroimaging in dementia: The NeuroSKILL project**

The NeuroSKILL project is an EU funded project involving Bangor University, Trinity College Dublin and University College Dublin. The project aims to develop training to increase capacity, knowledge and expertise regarding neuroimaging in dementia/Alzheimer’s Disease (AD) within North Wales and the East of Ireland. The aim of this questionnaire is to help us better understand the use of neuroimaging within these regions. We will use this information to develop training programs on neuroimaging in dementia. We will also use this information to write a report on the usability and accessibility of neuroimaging within these regions.

Any personal information you provide will be kept strictly confidential. If you choose to provide us with your contact details then this will be stored separately from your questionnaire. Please read all the questions carefully. You do not have to answer any questions that you do not wish to answer; you can just leave those questions blank.

**By completing this questionnaire you are consenting to take part in this study.**

**Section A.**

1. **What is your job title:**………………………………………………………………………………
2. **Are you:** Male Female
3. **Do you work in:** North Wales Ireland

County: ………………………………………………………

1. **Do you work in:**

General Medical Scheme/Public Health Service Private

Mixed Other, please state………………………………………………….

1. **In which year did you qualify as a practitioner?**...........................................
2. **How long have you been in your current job?**............................................
3. **Approximately what percentage of your clients is over 65 years of age?**

0 - 24% 25 - 49% 50 - 74% 75 - 100**%**

1. **If you are a GP please answer the following questions (if not go to Section B):**
2. **Please indicate your practice type:** Group Single
3. **Please specify your practice location:** Urban Rural Semi-Rural

**Section B.**

1. **Are you involved in the diagnosis of dementia/AD/mild cognitive impairment (MCI)?**

Yes No

1. **How would you rate the capabilities within your Health Board/HSE region in the diagnosis of dementia/AD/MCI?**

Excellent Good Fair Poor

1. **What would improve the rate of diagnosis of dementia/AD/MCI in your area?**

……………………………………………………………………………………………………………………………………………………………………………………………………………………………………………………………………………………………………
…………………………………………………………………………………………………………………………………………………

1. **Please rank what types of neuroimaging are available to you (with 1= most available, please leave blank any which are not available).**

MRI HMPAO SPECT FP-CIT SPECT FDG-PET or FDG-PET/CT CT

1. **In your opinion, how important is neuroimaging for the diagnosis of dementia/AD as opposed to other diagnostic tests?**

Very important Important Somewhat important Unimportant

**Section C.**

1. **Do you refer people with suspected MCI for neuroimaging?**

Yes No

1. **Do you refer people with suspected dementia/AD for neuroimaging?**

Yes No (If you answered **NO** to both questions 14 and 15 go to **Section D**)

1. **Why do you refer people with suspected MCI or dementia/AD for neuroimaging? (please tick all that apply)**

To establish a differential diagnosis To establish the sub-type of dementia

To rule out other causes/diseases Clinical guidelines (e.g. NICE)

Person under the age of 65

Other, please state:…………………………………………………………………………………………….......................

1. **Are you aware of any dementia specific protocols for referrals for neuroimaging?**

Yes No

1. **What types of neuroimaging do you refer to? (please tick all that apply)**

MRI HMPAO SPECT FP-CIT SPECT FDG-PET or FDG-PET/CT
CT None

1. **How confident are you in your ability to choose the most appropriate type of neuroimaging?**

Very confident Somewhat confident Not very confident Not at all confident

1. **Are your requests for neuroimaging ever refused?**

Never Rarely Sometimes Quite frequently

1. **If your requests for neuroimaging are refused, why are they refused?**

……………………………………………………………………………………………………………………………………….

1. **Are there types of neuroimaging you would like have access to but currently do not?**

No Yes, Please state...............................................................................................

1. **What factors would influence your decision not to request a scan (e.g. waiting times, lack of private health insurance?)**

..................................................................................................................................................................................................................................................................................................................................................................................................................................................................

**Section D.**

1. **Do you have access to reports on neuroimaging investigations about patients with suspected dementia/AD/MCI?**

Yes No (If **NO** go to **Section E**)

1. **How helpful is this information to you?**

Very helpful Helpful Unhelpful Very unhelpful

1. **How understandable is this information?**

Excellent Good Fair Poor

1. **If you are involved in explaining the report to patients and their family members, how easy is it to explain the information to them?**

Very Easy Easy Difficult Very difficult N/A

**Section E.**

1. **Have you ever received training on neuroimaging?** Yes No
2. **Have you ever received dementia-specific training?** Yes No
3. **How confident are you in your understanding of neuroimaging in dementia/AD?**

Very confident Somewhat confident Not very confident Not at all confident

1. **Would you like more information about the different types of neuroimaging available?**

Yes No

1. **Please indicate how important it is for you to further your knowledge about**

**a) Neuroimaging**Very important Important Somewhat important Unimportant

**b) Neuroimaging in dementia/AD**
Very important Important Somewhat important Unimportant

1. **Would you be interested in online training, study days or courses on the following:**
   **a) Neuroimaging**Yes No Unsure

   **b) Neuroimaging in dementia/AD**
   Yes No Unsure

Please comment:

…………………….…………………………………………………………………………………………………………………………………………………………………………………………………………………………………………………………………………………………………….

…………………….…………………………………………………………………………………………………………………………………………………………………………………………………………………………………………………………………………………………………….

Any other comments you would like to make about the use of neuroimaging in dementia/AD:

………….……………………………………………………………………………………………………………………………………………………………………………………………………………………………………………………………………………………………………………….

…………………….…………………………………………………………………………………………………………………………………………………………………………………………………………………………………………………………………………………………………….

**Thank you for completing this questionnaire**
